# Effects of Replacing Soybean Meal with Fermented Rapeseed Meal on Growth Performance, Meat Quality, Intestinal Morphology and Short-Chain Fatty Acid Production in Finishing Pigs

**DOI:** 10.3390/ani16132094

**Published:** 2026-07-06

**Authors:** Hao Cheng, Jiang Zhu, Enkai Li, Bie Tan, Yulong Yin, Xiaokang Ma, Qiang Tu

**Affiliations:** 1 Institute of Synthetic Biology Industry, Hunan University of Arts and Science, Changde 415000, China; chenghao19970316@163.com; 2 College of Animal Science and Technology, Hunan Agricultural University, Changsha 410128, China; 3 Department of Animal Sciences, Purdue University, West Lafayette, IN 47907, USA; 4 Yuelushan Laboratory, Changsha 410128, China; 5 Helmholtz International Lab for Anti-Infectives, State Key Laboratory of Microbial Technology, Shandong University, Qingdao 266237, China

**Keywords:** fermented rapeseed meal, finishing pigs, meat quality, performance, intestinal morphology

## Abstract

The objective of this study was to evaluate the effects of fermented rapeseed meal (FRSM) replacing soybean meal (SBM) on growth performance, carcass traits, meat quality, serum biochemical parameters, and intestinal morphology in finishing pigs. Fifty healthy barrows (Duroc × Landrace × Yorkshire, DLY) were randomly assigned to five groups (n = 10): group A (control, soybean meal-based diet with 0% FRSM) and groups B, C, D, and E with 2.8%, 5.6%, 8.4%, and 11.2% FRSM inclusion, respectively. FRSM inclusion had no significant effect on growth performance. Compared with group A, group C showed greater bone weight and backfat thickness (*p* < 0.05), whereas group D had a lower dressing percentage (*p* < 0.05). Meanwhile, group D showed improved pressing loss and increased levels of arachidonic acid and α-linolenic acid (*p* < 0.05). Serum analysis revealed reduced malondialdehyde and elevated alanine, methionine, lysine, and cysteine concentrations in group D (*p* < 0.05). Jejunal villus height and villus height-to-crypt depth ratio were significantly increased in groups D and E, indicating improved intestinal morphology. However, group E reduced colonic acetic acid concentration. Adding 8.4% FRSM improved fatty acid composition, meat quality, and intestinal structure without compromising growth performance, suggesting that FRSM is a viable and sustainable alternative protein source in finishing pig diets.

## Simple Summary

Soybean meal is widely used as a protein source in pig feed, but its increasing cost has encouraged the search for alternative ingredients. In this study, fermented rapeseed meal (FRSM) was used to replace different levels of soybean meal in finishing pig diets. Fifty pigs were assigned to five dietary treatments with 0%, 2.8%, 5.6%, 8.4%, or 11.2% FRSM inclusion. The results showed that FRSM did not negatively affect pig growth performance. Importantly, dietary 8.4% FRSM improved meat quality, increased beneficial fatty acids, reduced oxidative stress, while altering serum amino acid profiles. Overall, fermented rapeseed meal can serve as a sustainable and cost-effective alternative protein source in finishing pig diets, with 8.4% inclusion showing the greatest benefits.

## 1. Introduction

The imbalance between the supply and demand of protein feed resources has become increasingly pronounced, particularly in developing countries, largely due to the global shortage and escalating cost of soybean meal (SBM), the primary plant-based protein source in animal nutrition. This situation has driven extensive efforts to identify sustainable and cost-effective alternatives to SBM [1,2,3].

Rapeseed meal (RSM), the second most abundant plant protein source worldwide, has attracted considerable attention as a potential substitute because of its relatively balanced amino acid profile [4]. Compared with SBM, RSM contains higher levels of sulfur-containing amino acids but is relatively deficient in lysine [5]. However, its application in animal diets is limited by the presence of glucosinolates and other anti-nutritional factors, which are associated with adverse physiological effects, including thyroid dysfunction, hepatorenal hypertrophy, impaired growth, and increased mortality [6]. In addition, RSM generally has higher crude fiber content and lower protein concentration than SBM, thereby requiring appropriate processing to improve its nutritional value and digestibility [7].

Biotechnological approaches, such as microbial fermentation and enzymatic hydrolysis, have been widely applied to enhance the nutritional quality of plant protein sources. Fermentation can effectively reduce glucosinolate content [6], while combined enzymatic and microbial treatments further degrade fiber and anti-nutritional compounds, thereby improving nutrient digestibility [8,9]. Moreover, fermentation has been shown to enhance the palatability and modify the volatile compound profile of feed ingredients [10,11,12]. However, several studies have demonstrated that fermented rapeseed meal can improve nutrient utilization and intestinal function in nursery and growing pigs. Information regarding the optimal replacement level of soybean meal with fermented rapeseed meal in finishing pigs remains limited, particularly with respect to carcass traits, meat quality, intestinal morphology, serum amino acid profiles, and short-chain fatty acid production. Therefore, further studies are required to evaluate the feasibility and appropriate inclusion level of FRSM in finishing pig diets.

Accordingly, the present study evaluated the effects of replacing soybean meal with fermented rapeseed meal on growth performance, carcass characteristics, meat quality, serum biochemical parameters, serum amino acid profiles, intestinal morphology, and short-chain fatty acid production in finishing pigs. We hypothesized that partial replacement of soybean meal with fermented rapeseed meal would improve jejunal morphology and meat quality without impairing growth performance, thereby providing an effective and sustainable alternative protein source for finishing pig diets.

## 2. Materials and Methods

### 2.1. Preparation of Fermented Rapeseed Meal

Rapeseed meal was procured from Jiexin Grain and Oil Processing Co., Ltd. (Changde, China). FRSM was produced via solid-state fermentation using a microbial consortium comprising *Bacillus subtilis*, *Saccharomyces cerevisiae*, and *Lactobacillus plantarum*, supplemented with cellulase, pectinase, and protease (Beijing Challenge International Trade Co., Ltd. Beijing, China). The substrate was adjusted to a 50% moisture content, inoculated with microorganisms and enzymes, and incubated at 40 °C for 48 h. As shown in Table 1, fermentation increased crude protein and amino acid contents while reducing fiber fractions and glucosinolate concentration, suggesting an improvement in the nutritional quality of rapeseed meal. Crude ash was determined by combustion in a muffle furnace (SX2-4-10NP, Shanghai Jingke Scientific Instrument Co., Ltd., Shanghai, China) at 550 °C (GB/T 6438-2007, China National Standard, 2007) [13]. Crude protein (CP) was determined by the Kjeldahl method (GB/T 6432-2018, China National Standard, 2018) using an automatic Kjeldahl analyzer (K9860, Hanon Instruments, Ji’nan, China) [14]. Amino acids were analyzed by GB/T 18246-2019 (China National Standard, 2019) using an Amino Acid Analyzer (L-8900-1, Hitachi High-Tech Corp., Dalian, China) [15].

### 2.2. Animals and Experimental Design

A total of 50 Duroc × Landrace × Yorkshire (DLY) barrows (initial body weight = 68.63 ± 3.54 kg, an average age of 120 ± 5 days) were randomly assigned to five dietary treatments (n = 10 per group; one pig per pen) for 42 days in a completely randomized design. Treatments included: Control (A): soybean meal-based diet; B: 2.8% FRSM; C: 5.6% FRSM; D: 8.4% FRSM; E: 11.2% FRSM. The nutrient composition of SBM and FRSM is provided in Table 2. All diets met or exceeded NRC (2012) requirements [16]. Pigs were individually housed in pens (1.5 × 2.0 m) within a temperature-controlled facility (24 ± 2 °C; RH 60 ± 5%) with slatted plastic floors, ad libitum access to feed and water, and automatic feeders and nipple drinkers.

### 2.3. Sample Collection

On the 21st day of the experiment, fresh fecal samples were collected from the finishing pig’s anus and stored at −80 °C for the detection of short-chain fatty acid content. Blood samples from the finishing pigs were collected through the anterior vena cava, which were centrifuged at 4000 rpm for 15 min to separate the serum, and then stored at −80 °C for the detection of serum biochemical indicators and amino acids. Over 500 g of the longissimus dorsi muscle samples were collected for meat quality and fatty acid assessment.

### 2.4. Growth Performance

Individual body weights were recorded at the beginning and end of the trial following overnight fasting. Feed intake was recorded daily, and all data were collected using calibrated electronic scales (±0.01 kg). ADFI (average daily feed intake, ADFI) = Total feed intake/(Number of animals × Trial period); ADG (average daily gain, ADG) = Total weight gain/(Number of animals × Trial period); Gain: feed = ADG/ADFI.

### 2.5. Carcass Characteristics

Pigs were fasted for 12 h with free access to water prior to slaughter. Animals were electrically stunned (250 V, 0.5 A for 5 s) and subsequently exsanguinated according to commercial slaughter procedures. Carcasses were scalded, dehaired, eviscerated, and split longitudinally. Backfat thickness was measured at three anatomical locations, including the first rib, last rib, and last lumbar vertebra, and the average value was used for statistical analysis. After slaughter, carcasses were split longitudinally. Carcass weight was recorded after evisceration, and dressing percentage was calculated as carcass weight relative to final body weight. Carcass length and diagonal length were measured from the first rib to the pubic bone and ischial tuberosity, respectively. The eye muscle area (longissimus dorsi) was determined at the 10th rib. Bone weight, tare weight, and leanness were obtained based on carcass dissection, with leanness expressed as lean meat percentage of carcass weight. The leg-to-hip ratio was calculated as hind leg weight relative to carcass weight.

### 2.6. Meat Quality

Muscle pH was measured at 24 h postmortem using a portable pH meter (HI99161, Hanna Instruments, Roma, Italy). Meat color (L*, a*, b*) was assessed with a Minolta CR-410 spectrophotometer (Konica Minolta, Tokyo, Japan). Drip loss was determined using longissimus dorsi samples (3 cm × 2 cm × 1 cm). Initial weight (W1) was recorded, and samples were suspended in sealed plastic bags at 4 °C for 24 h. After blotting surface moisture, the final weight (W2) was recorded, and drip loss was calculated as (W1 − W2)/W1 × 100%. Pressing loss. Samples of the longissimus dorsi muscle were cut into 1 cm-thick sections using a circular sampler. Each sample was weighed and recorded. The samples were then placed between 48 layers of filter paper, with the same number of filter paper layers placed on top. Pressure was applied using a standardized instrument. After pressing, the samples were reweighed, and the pressing loss was calculated. Shear force was measured on muscle samples (≥10 cm × 6 cm × 6 cm) with removed fat and fascia after storage at 4 °C for 24 h, using a texture analyzer. Intramuscular fat (IMF) was determined from freeze-dried (~100 g) samples using Soxhlet extraction. Cooking loss was measured on ~50 g psoas major samples. After weighing (W4), samples were cooked at 100 °C for 10 min, hung for 5 min, and reweighed (W5), calculated as (W4 − W5)/W4 × 100%. Meat quality parameters were determined following the methodology previously described by Yang et al. (2026) [17].

### 2.7. Fatty Acid and Amino Acid Composition

Lipids were extracted and converted to fatty acid methyl esters (FAMEs), which were subsequently analyzed using a gas chromatograph (Agilent 6890N, Agilent Technologies, Chicago, IL, USA) equipped with a flame ionization detector and a DB-23 capillary column (60 m × 0.25 mm × 0.25 μm). Helium was used as the carrier gas, and the injector and detector temperatures were maintained at 250 °C and 280 °C, respectively.

Serum free amino acids were quantified using an automatic amino acid analyzer (L-8900, Hitachi, Tokyo, Japan). Serum samples were deproteinized with sulfosalicylic acid, centrifuged, filtered through a 0.22 μm membrane filter, and analyzed by ion-exchange chromatography with post-column ninhydrin derivatization according to the manufacturer’s protocol.

### 2.8. Serum Biochemical Analysis

Serum levels of IgG, IgM, IgA, SOD, T-AOC, and MDA were quantified using commercial ELISA kits (Jiangsu Meimian Industrial Co., Ltd. Nanjing, China).

### 2.9. Intestinal Morphology

Jejunal samples were collected approximately 10 cm distal to the ligament of Treitz, whereas ileal samples were collected approximately 10 cm proximal to the ileocecal junction. Tissue samples were fixed in 4% paraformaldehyde for 24 h, dehydrated through a graded ethanol series, embedded in paraffin, sectioned at 3 μm, and stained with hematoxylin and eosin. Morphological measurements were performed by an investigator blinded to treatment allocation. Six typical villi were selected from each section, and the villi height (VH) and crypt depth (CD) were measured using the Case Viewer system (version 2.3), and the ratio of villi to crypt (VH/CD) was calculated.

### 2.10. Statistical Analysis

Data were analyzed using one-way ANOVA in SPSS 27.0 with Tukey’s test for post hoc comparisons. Individual pigs were considered the experimental unit. Data normality and homogeneity of variance were assessed using the Shapiro–Wilk test and Levene’s test, respectively. Orthogonal polynomial contrasts were conducted to evaluate linear and quadratic responses to increasing dietary FRSM inclusion levels, and these contrasts were specified a priori according to the experimental design. Results are expressed as means ± SEM, with significance defined at *p* < 0.05 and trends at 0.05 ≤ *p* ≤ 0.10.

## 3. Results

### 3.1. Growth Performance

As shown in Table 3, no significant differences (*p* > 0.05) were observed in ADG, ADFI, or FCR among treatment groups. However, pigs fed diet D exhibited numerically higher ADG and Gain:feed, and lower ADFI than other groups.

### 3.2. Carcass Traits

As shown in Table 4, bone weight and backfat thickness were significantly higher in group C than in group A (*p* < 0.05). The dressing percentage in group D was significantly reduced relative to group A (linear *p* < 0.05).

### 3.3. Meat Quality and Fatty Acid Composition

Table 5 shows that FRSM inclusion had no significant effects on lightness, yellowness, drip loss, intramuscular fat, cooking loss, or shear force (*p* > 0.05). A significant quadratic effect was observed for ultimate pH (*p* < 0.05), with higher pH values in groups C and D. Additionally, pressing loss was significantly lower in group D compared with the control group A (*p* < 0.05). Redness (a*) was significantly higher in groups D and E than in group A (*p* < 0.05).

As presented in Table 6, group D had markedly greater concentrations of arachidonic acid (C20:4 n-6) and α-linolenic acid (C18:3 n-3) (*p* < 0.05), suggesting an enhanced polyunsaturated fatty acid profile.

### 3.4. Serum Biochemical Parameters

Serum alanine, methionine, lysine, and cysteine concentrations were significantly elevated in group D compared with group A (linear *p* < 0.05) in Table 7. Table 8 indicates that serum IgG, IgA, IgM, SOD, and T-AOC were unaffected (*p* > 0.05). MDA levels were significantly reduced in group D (*p* < 0.05).

### 3.5. Intestinal Morphology

As shown in Table 9, jejunal villus height increased linearly with higher FRSM inclusion (*p* < 0.05). Groups D and E exhibited greater villus height than group A, and group D had the highest VH/CD ratio (*p* < 0.05).

### 3.6. Short-Chain Fatty Acids

As shown in Table 10, ileal SCFA (short-chain fatty acids) concentrations did not differ among treatments (*p* > 0.05). However, colonic acetic acid concentrations were significantly reduced in group E (*p* < 0.05).

## 4. Discussion

The application of rapeseed meal (RSM) in swine diets has long been constrained by anti-nutritional factors, particularly glucosinolates, which negatively affect animal health and growth performance [7]. In recent years, microbial fermentation has emerged as an effective strategy to detoxify RSM and improve its nutritional value. Previous studies have demonstrated that fermentation with *Saccharomyces cerevisiae*, *Bacillus subtilis*, or *Lactobacillus* can significantly reduce glucosinolate content, degrade fiber fractions, and increase the bioavailability of nutrients and small peptides [18,19]. Shi et al. (2016) demonstrated that *Aspergillus niger*-fermented rapeseed meal significantly improved average daily gain (ADG) and feed conversion efficiency in growing pigs, enhanced the apparent digestibility of dry matter, crude protein, calcium, and phosphorus, and reduced serum aspartate aminotransferase (AST) levels [20]. Shuai et al. (2023) also reported that, compared with non-fermented rapeseed meal, fermented rapeseed meal significantly increased ADG and final body weight in growing pigs, elevated the villus height to crypt depth (VH/CD) ratio in the small intestine [21]. Consistent with these findings, the present study showed that FRSM had no adverse effects on growth performance, suggesting that soybean meal can be partially replaced by FRSM without compromising nutrient utilization efficiency. Although no significant differences were observed, pigs in group D showed numerically improved ADG and feed efficiency, which may indicate a trend toward enhanced nutrient utilization. This improvement is likely associated with fermentation-induced degradation of anti-nutritional factors and increased digestibility of proteins and peptides, thereby improving intestinal nutrient availability.

Carcass and meat quality parameters are crucial indicators of production efficiency and consumer acceptability. Yang et al. (2026) demonstrated that FRSM significantly improved meat quality and lipid metabolism in finishing pigs compared with RSM [17]. Cheng et al. (2024) reported that partial substitution of SBM with FRSM enhanced carcass traits without impairing meat quality [22]. Similarly, our findings revealed that moderate FRSM inclusion (5.6–8.4%) increased bone weight and backfat thickness, possibly due to enhanced lipid metabolism and mineral bioavailability. Unexpectedly, pigs receiving intermediate levels of FRSM exhibited increased bone weight and backfat thickness, accompanied by a slight reduction in dressing percentage. The biological mechanisms responsible for these responses remain unclear. These changes may reflect alterations in nutrient partitioning or individual variation rather than direct effects of FRSM itself.

Moreover, we found that the decreased pressing loss and enrichment of polyunsaturated fatty acids, particularly arachidonic acid and α-linolenic acid, in the 8.4% FRSM inclusion group suggest an improvement in both the nutritional value and sensory quality of pork. These changes may be linked to fermentation-derived bioactive compounds, such as phenolic substances and organic acids, which are known to regulate lipid metabolism and fatty acid deposition.

In the present study, the reduction in serum malondialdehyde (MDA) concentrations suggests reduced lipid peroxidation and improved oxidative status, while the increased circulating concentrations of alanine, methionine, lysine, and cysteine may reflect alterations in amino acid metabolism, absorption, or utilization. Fermentation-derived bioactive peptides and phenolic compounds may contribute to antioxidant defense by scavenging reactive oxygen species and regulating redox homeostasis. Improved serum amino acid profiles may also be linked to enhanced intestinal morphology and absorptive function.

Liu et al. (2026) [23] reported that replacing part of the SBM in the diet with FRSM can enhance growth performance, improve antioxidant capacity and immune function, and benefit intestinal morphology. Improved intestinal morphology, as evidenced by increased villus height and villus height-to-crypt depth ratio, may facilitate nutrient absorption and contribute to the elevated amino acid levels observed in circulation [23]. Consistent with previous reports, in the present study, intestinal morphology analysis demonstrated that FRSM improved jejunal villus height and the VH/CD ratio. These improvements indicate enhanced mucosal development and nutrient absorption capacity.

Although short-chain fatty acid (SCFA) levels in the ileum were unaffected, colonic acetate concentration decreased with the inclusion of FRSM levels. Microbial metabolites play pivotal roles in maintaining intestinal homeostasis and host physiology. SCFAs are important microbial metabolites involved in maintaining intestinal epithelial integrity and metabolic homeostasis [24]. The reduction in colonic acetate at excessive FRSM inclusion may reflect alterations in hindgut microbial fermentation patterns, potentially due to increased dietary fiber or substrate shifts. Similar responses have been observed in high-fiber fermented feed systems, where excessive inclusion levels can disrupt microbial fermentation balance [25,26]. This suggests that there is an optimal inclusion range for FRSM to maximize intestinal benefits without disturbing microbial metabolic stability. However, the underlying mechanisms remain unclear, as the present study did not comprehensively characterize the fermentation properties of FRSM, including the extent of glucosinolate degradation, residual anti-nutritional factors, and fermentation-derived bioactive compounds. Further studies are therefore needed to clarify the relationships between FRSM composition and its physiological effects in pigs.

## 5. Conclusions

In conclusion, dietary inclusion of fermented rapeseed meal did not impair growth performance and showed beneficial effects on meat quality, fatty acid composition, oxidative status, and intestinal morphology in finishing pigs. Among the tested inclusion levels, 8.4% FRSM appeared to provide the most favorable overall responses under the conditions of the present study. These findings suggest that FRSM may serve as a sustainable alternative protein source in finishing pig diets; however, further studies are required to determine the optimal inclusion level and clarify the underlying mechanisms.

## Figures and Tables

**Table 1:** Conventional nutrient analysis of FRSM and RSM (dry matter basis) ^1^.

Items	RSM	FRSM
Crude fat %	0.94	0.90
Crude Protein %	43.03	45.65
Crude fiber %	16.14	14.91
Neutral detergent fiber %	54.62	48.37
Acid detergent fiber %	43.70	36.55
Crude ash %	9.06	8.64
Amylum %	2.50	1.08
Calcium %	0.75	0.68
Phosphorus %	0.95	0.79
Thioside mg/g	4.13	2.98
Arginine	1.96	2.17
Histidine	1.20	1.30
Isoleucine	1.68	1.84
Leucine	3.04	3.35
Lysine	1.28	1.35
Methionine	0.82	0.91
Phenylalanine	1.70	1.84
Threonine	1.73	1.92
Tryptophan	0.45	0.34
Valine	2.15	2.43
Alanine	1.99	2.20
Aspartate	2.82	3.06
Cystine	0.84	0.92
Glutamate	7.91	8.79
Glycine	2.14	2.39
Proline	2.70	2.96
Serine	1.66	1.85
Tyrosine	0.99	1.10

^1^ RSM, rapeseed meal; FRSM, fermented rapeseed meal.

**Table 2:** Composition and nutrient levels of the basal diets (g/kg, as-fed basis).

Ingredients/Group	A	B	C	D	E
Corn	44.38	44.06	43.90	43.82	43.59
Brown rice	23.00	23.00	23.00	23.00	23.00
Rice bran meal	16.50	16.50	16.50	16.50	16.50
Soybean meal	12.00	9.00	6.00	3.00	0.00
FRSM	0.00	2.80	5.60	8.40	11.20
Soybean oil	1.15	1.55	1.85	2.15	2.50
CaPHO_4_	0.55	0.6	0.55	0.45	0.45
Limestone	0.99	0.98	1.00	1.00	1.00
NaCl	0.30	0.30	0.30	0.30	0.30
Lys	0.24	0.30	0.38	0.45	0.50
Met	0.01	0.01	0.01	0.01	0.01
Thr	0.07	0.08	0.09	0.09	0.10
Trp	0.01	0.02	0.02	0.03	0.05
TiO_2_	0.30	0.30	0.30	0.30	0.30
Premix ^1^	0.50	0.50	0.50	0.50	0.50
Nutrient composition ^2^					
NE MJ/kg	10.21	10.21	10.21	10.21	10.21
CP %	13.52	13.50	13.50	13.50	13.48
Ca %	0.56	0.57	0.58	0.56	0.57
Available P %	0.19	0.20	0.19	0.19	0.19
SID lys %	0.70	0.70	0.71	0.72	0.71
SID met%	0.21	0.21	0.22	0.22	0.22
SID Thr %	0.46	0.46	0.47	0.47	0.47
SID Trp %	0.13	0.13	0.12	0.12	0.13
Nutrients ^3^					
Dry matter %	88.12	88.56	88.51	88.34	88.62
Crude protein%	13.65	13.52	13.59	13.69	13.66
Crude fat %	3.39	3.91	3.92	3.98	4.27
GE, MJ/kg	16.77	16.76	16.89	16.46	17.12
Crude fiber %	4.23	3.59	4.63	3.94	3.43
Ash %	5.06	5.23	5.22	4.84	4.64
Neutral detergent fiber %	14.78	11.46	15.01	18.29	17.22
Acid detergent fiber %	3.27	1.80	4.80	6.14	6.59

^1^ Premix provides the following per kilogram of diet: Vitamin A (retinyl acetate), 32,500 IU; Vitamin B6 (pyridoxine hydrochloride), 8 mg; Vitamin D3 (cholecalciferol), 10,000 IU; Vitamin B12 (cyanocobalamin), 0.075 mg; Vitamin E (DL-α-tocopheryl acetate), 80 IU; D-Biotin, 0.6 mg; Vitamin K3 (menadione sodium bisulfite), 10 mg; Folic acid, 5 mg; Vitamin B1 (thiamine mononitrate), 10 mg; Vitamin B2 (riboflavin), 25 mg; Niacin, 100 mg; D-Pantothenic acid, 50 mg; Choline, 1,600 mg; Mn (manganese sulfate monohydrate), 300 mg; Fe (ferrous sulfate monohydrate), 600 mg; Zn (zinc sulfate monohydrate), 300 mg; Cu (copper sulfate pentahydrate), 500 mg; Zeolite powder, 714.29 mg. ^2^ Nutritional levels are calculated values. ^3^ Nutrient composition represents measured values. A, control diet containing soybean meal without fermented rapeseed meal (FRSM); B, diet containing 2.8% FRSM; C, diet containing 5.6% FRSM; D, diet containing 8.4% FRSM; and E, diet containing 11.2% FRSM. FRSM, fermented rapeseed meal; SID, standardized ileal digestibility. NE, net enrgy; GE, gross energy.

**Table 3:** Effects of FRSM on the growing performance of finishing pigs.

Items	A	B	C	D	E	SEM	*p*-Value		
							Anova	Linearly	Quadratic
Initial weight (kg)	68.90	68.05	68.75	68.80	68.40	1.09	0.98	0.94	0.95
Final weight (kg)	107.20	105.95	107.15	107.50	105.95	2.15	0.97	0.89	0.86
Average daily gain (kg)	0.85	0.84	0.85	0.86	0.83	0.04	0.99	0.89	0.79
Average daily feed intake (kg)	2.65	2.69	2.72	2.64	2.66	0.06	0.92	0.88	0.56
Gain:feed	0.31	0.31	0.31	0.32	0.31	0.01	0.94	0.79	0.98

Data are the arithmetic means of ten (n = 10) observations. SEM, standard error of the mean; Gain:feed, weight gain/feed intake. FRSM, fermented rapeseed meal.

**Table 4:** Effects of FRSM on the carcass performance of finishing pigs.

Items	A	B	C	D	E	SEM	*p*-Value		
							Anova	Linearly	Quadratic
Tare weight (kg)	4.88	4.75	5.22	4.92	4.87	0.22	0.64	0.85	0.47
Bone weight (kg)	10.85	11.00	11.83	10.52	11.10	0.21	0.01	0.98	0.12
Carcass weight (kg)	77.41	78.89	82.33	80.84	81.58	1.32	0.09	0.02	0.21
Dressing percentage (%)	74.97	75.88	74.33	73.19	74.81	0.50	0.02	0.07	0.34
Leg-to-hip ratio (%)	31.24	31.63	31.98	31.62	31.73	0.49	0.88	0.54	0.50
Carcass length (cm)	99.67	97.83	97.17	99.67	96.50	1.37	0.38	0.31	0.92
Carcass diagonal length (cm)	86.17	85.67	85.17	87.33	85.83	1.11	0.71	0.78	0.87
Backfat thickness (mm)	29.47	30.79	33.63	31.48	33.62	1.00	0.03	0.01	0.38
Eye muscle area (cm^2^)	40.80	41.19	45.38	45.34	42.92	2.02	0.35	0.20	0.21
Leanness (%)	55.14	54.68	56.04	54.66	54.18	1.52	0.93	0.69	0.63

Data are the arithmetic means of ten (n = 10) observations. SEM, standard error of the mean. FRSM, fermented rapeseed meal.

**Table 5:** Effects of FRSM on the meat quality of finishing pigs.

Items	A	B	C	D	E	SEM	*p*-Value		
							Anova	Linearly	Quadratic
pH	5.43	5.55	5.63	5.64	5.54	0.04	0.04	0.06	0.01
Lightness	43.13	43.17	43.47	42.16	44.26	1.00	0.69	0.70	0.51
Redness	6.30	6.81	7.02	7.64	7.37	0.30	0.04	0.01	0.32
Yellowness	5.74	6.40	6.41	6.26	6.68	0.30	0.29	0.08	0.57
Marbling	3.25	3.33	3.17	3.08	2.92	0.27	0.84	0.29	0.68
Drip loss (%)	4.87	4.68	4.19	4.20	3.52	0.43	0.24	0.03	0.77
Pressing loss (%)	31.43	30.93	29.86	26.06	30.15	1.18	0.03	0.06	0.16
IMF (%)	2.22	2.27	2.32	2.41	2.25	0.19	0.97	0.78	0.61
Cooking loss (%)	27.53	27.33	27.88	29.84	29.77	1.28	0.47	0.10	0.73
Shear force (N)	65.67	57.30	61.26	66.86	68.39	6.04	0.69	0.44	0.35

Data are the arithmetic means of ten (n = 10) observations. SEM, standard error of the mean; IMF, intramuscular fat. FRSM, fermented rapeseed meal.

**Table 6:** Effects of FRSM on the muscle’s fatty acid of finishing pigs.

Items	A	B	C	D	E	SEM	*p*-Value		
							Anova	Linearly	Quadratic
Methyl laurate (C12:0)	0.10	0.08	0.09	0.09	0.08	0.01	0.44	0.18	0.70
Myristic acid (C14:0)	1.23	1.32	1.36	1.33	1.32	0.06	0.52	0.27	0.20
Heneicosanoic acid (C15:0)	0.05	0.05	0.04	0.04	0.04	0.01	0.46	0.08	0.94
Palmitic acid (C16:0)	25.64	25.80	25.64	25.67	25.52	0.39	0.99	0.76	0.77
Palmitoleic acid (C16:1)	2.81	2.51	2.87	2.61	2.93	0.14	0.24	0.47	0.27
Margaric acid (C17:0)	0.26	0.24	0.21	0.21	0.23	0.02	0.41	0.20	0.20
Heptadecenoic acid (C17:1)	0.20	0.19	0.18	0.16	0.21	0.02	0.65	0.89	0.24
Stearic acid (C18:0)	14.66	15.31	14.98	15.07	14.56	0.40	0.68	0.73	0.23
Oleic acid (C18:1n-9c)	39.45	39.05	41.06	39.24	41.32	1.02	0.39	0.24	0.77
Linoleic acid (C18:2n-6c)	9.94	10.41	9.16	10.50	9.62	0.62	0.54	0.78	0.97
γ-Linolenic acid (C18:3n-6)	0.13	0.11	0.09	0.07	0.06	0.02	0.13	0.01	0.80
α-Linolenic acid (C18:3n-3)	0.29	0.43	0.41	0.45	0.46	0.03	<0.01	<0.01	0.07
Methyl heineicosanoate (C21:0)	0.05	0.05	0.05	0.05	0.07	0.03	0.94	0.70	0.57
Arachidic acid (C20:0)	0.25	0.24	0.26	0.25	0.23	0.02	0.86	0.51	0.58
Eicosenoic acid (C20:1)	0.89	0.82	0.84	0.84	0.80	0.04	0.49	0.15	0.77
Eicosadienoic acid (C20:2)	0.34	0.37	0.34	0.36	0.29	0.03	0.33	0.24	0.15
Dihomo-γ-linolenic acid (C20:3n-6)	0.32	0.27	0.23	0.27	0.19	0.04	0.15	<0.01	0.79
Eicosatrienoic acid (C20:3n-3)	0.06	0.05	0.06	0.04	0.02	0.01	0.16	0.02	0.50
Arachidonic acid (C20:4n-6)	0.01	0.63	1.48	1.92	1.60	0.29	<0.01	<0.01	<0.01
Behenic acid (C22:0)	0.15	0.11	0.09	0.11	0.10	0.02	0.36	0.12	0.27
SFA	41.85	41.60	42.44	43.12	42.00	0.72	0.60	0.43	0.49
MUFA	44.34	44.59	44.77	42.43	45.24	1.05	0.40	0.92	0.51
PUFA	10.09	10.70	9.90	10.88	9.95	0.57	0.64	0.96	0.55
n-6:n-3	28.83	22.89	22.46	25.60	22.82	1.61	0.05	0.08	0.12

Data are the arithmetic means of ten (n = 10) observations. SEM, standard error of the mean; SFA, saturated fatty acids; MUFA, monounsaturated fatty acids; PUFA, polyunsaturated fatty acids. FRSM, fermented rapeseed meal.

**Table 7:** Effects of FRSM on the serum free amino acid composition of finishing pigs.

Items	A	B	C	D	E	SEM	*p*-Value		
							Anova	Linearly	Quadratic
Asp	5.77	4.40	4.46	6.06	4.59	1.09	0.71	0.84	0.74
Thr	38.08	37.72	45.25	54.70	39.46	4.39	0.06	0.17	0.11
Ser	11.04	11.07	12.38	16.13	11.20	1.69	0.20	0.33	0.25
Glu	42.99	34.85	43.24	47.68	36.45	4.89	0.35	0.98	0.59
Gly	63.81	66.70	58.23	61.73	60.37	4.69	0.75	0.43	0.85
Ala	46.16	36.32	44.37	55.59	42.07	3.86	0.03	0.37	0.77
Cys	12.26	11.65	14.00	16.34	14.64	0.90	0.01	<0.01	0.52
Val	34.09	33.32	45.37	49.56	40.43	4.72	0.10	0.07	0.18
Met	5.00	4.42	5.18	6.60	5.46	0.34	<0.01	0.01	0.73
Ile	11.35	11.30	8.14	7.85	10.12	1.06	0.07	0.09	0.07
Leu	24.78	23.85	33.10	32.60	31.59	4.96	0.53	0.17	0.60
Tyr	18.61	16.46	21.51	23.83	19.31	2.15	0.18	0.21	0.36
Phe	13.10	11.78	21.50	25.97	15.88	3.67	0.06	0.10	0.11
Lys	25.78	25.10	37.46	49.89	33.53	4.91	0.01	0.02	0.10
His	14.44	12.47	24.65	27.88	15.08	4.97	0.15	0.30	0.12
Trp	19.21	16.93	25.48	26.96	16.69	3.39	0.13	0.65	0.08
Arg	30.56	30.08	34.82	37.94	26.58	3.80	0.28	0.99	0.12
Pro	23.78	18.15	22.74	28.16	20.07	2.35	0.06	0.73	0.65
EAA	216.4	206.97	280.94	319.95	234.83	28.70	0.06	0.11	0.10
NEAA	224.42	199.59	220.95	255.52	208.69	16.42	0.19	0.64	0.62
TAA	440.82	406.57	501.89	575.47	443.51	41.31	0.07	0.20	0.18

Data are the arithmetic means of ten (n = 10) observations. SEM, standard error of the mean; EAA, essential amino acid; NEAA, non-essential amino acid; TAA, total amino acid. FRSM, fermented rapeseed meal.

**Table 8:** Effects of FRSM on the serum parameters of finishing pigs.

Items	A	B	C	D	E	SEM	*p*-Value		
							Anova	Linearly	Quadratic
SOD (U/mL)	65.99	68.84	73.71	74.42	79.36	3.44	0.10	0.01	0.99
MDA (nmol/mL)	2.34	1.74	1.57	1.46	1.43	0.18	0.01	<0.01	0.09
T-AOC (U/mL)	5.05	5.17	5.23	5.36	5.49	0.12	0.14	0.01	0.86
IgA (mg/mL)	1.65	1.41	1.51	1.76	1.57	0.14	0.51	0.69	0.66
IgG (mg/mL)	12.18	11.93	12.70	13.27	13.02	0.37	0.10	0.02	0.89
IgM (mg/mL)	2.30	2.21	2.34	2.46	2.56	0.17	0.63	0.17	0.55

Data are the arithmetic means of ten (n = 10) observations. SEM, standard error of the mean; SOD, superoxide dismutase; MDA, malondialdehyde; T-AOC, total antioxidant capacity; IgA, immunoglobulin A; IgG, immunoglobulin G; IgM, immunoglobulin M.

**Table 9:** Effects of FRSM on the intestinal morphology of finishing pigs.

Items	A	B	C	D	E	SEM	*p*-Value		
							Anova	Linearly	Quadratic
Jejunum									
Villus height (μm)	551.70	576.40	553.70	592.48	589.55	9.17	0.01	0.01	0.86
Crypt depth (μm)	442.75	456.05	420.52	414.72	468.87	11.45	0.01	0.77	0.02
VH/CD	1.25	1.27	1.32	1.4	1.26	0.04	0.02	0.16	0.04
Ileum									
Villus height (μm)	498.97	526.78	469.57	458.15	488.60	18.40	0.12	0.14	0.47
Crypt depth (μm)	367.68	356.57	323.78	289.85	325.55	27.83	0.34	0.10	0.39
VH/CD	1.39	1.53	1.49	1.62	1.52	0.10	0.57	0.27	0.39

Data are the arithmetic means of ten (n = 10) observations. SEM, standard error of the mean. VH/CD, Villus height/crypt depth. FRSM, fermented rapeseed meal.

**Table 10:** Effects of FRSM on short-chain fatty acids in intestinal contents of finishing pigs.

Items	A	B	C	D	E	SEM	*p*-Value		
							Anova	Linearly	Quadratic
Ileum									
Acetate	595.60	478.80	603.90	384.40	517.00	109.03	0.61	0.47	0.71
Propionate	301.31	232.06	283.11	174.79	247.56	36.55	0.17	0.17	0.37
Isobutyrate	23.25	30.26	37.22	14.12	26.19	10.88	0.65	0.77	0.63
Butyrate	131.55	97.90	135.93	81.77	117.14	32.70	0.74	0.67	0.71
Isovalerate	22.72	41.25	38.21	30.62	35.74	4.99	0.12	0.34	0.11
Valeric acid	19.23	36.25	39.99	20.68	29.43	21.89	0.95	0.95	0.63
TVFA	1093.70	916.50	1138.30	706.40	973.10	169.49	0.43	0.41	0.72
Colon									
Acetate	1774.40	1585.50	1625.10	1380.10	1160.40	125.18	0.02	<0.01	0.47
Propionate	791.81	702.97	752.59	604.48	585.52	56.02	0.07	0.01	0.79
Isobutyrate	60.86	50.50	61.31	59.42	66.15	5.52	0.40	0.28	0.31
Butyrate	438.07	339.78	382.82	322.62	292.76	46.34	0.24	0.05	0.85
Isovalerate	102.72	81.46	100.20	98.84	112.61	9.90	0.31	0.25	0.19
Valeric acid	75.06	58.79	72.92	67.74	85.49	7.45	0.18	0.22	0.10
TVFA	3242.92	2819.00	2994.94	2533.20	2302.93	227.56	0.06	0.01	0.77

Data are the arithmetic means of ten (n = 10) observations. SEM, standard error of the mean; TVFA = Total volatile fatty acid. FRSM, fermented rapeseed meal.

## Data Availability

The original contributions presented in this study are included in the article. Further inquiries can be directed to the corresponding author.
